# (*E*)-*N*′-(5-Bromo-2-hy­droxy­benzyl­idene)-3-methyl­benzohydrazide

**DOI:** 10.1107/S1600536811025426

**Published:** 2011-07-02

**Authors:** Hai-Chang Guo, Shi-Yong Liu, Xiao-Ling Wang

**Affiliations:** a College of Chemistry & Pharmacy, Taizhou University, Taizhou Zhejiang 317000, People’s Republic of China; b Department of Chemistry, Liaoning Normal University, Dalian 116029, People’s Republic of China

## Abstract

In the title mol­ecule, C_15_H_13_BrN_2_O_2_, an intra­molecular O—H⋯N hydrogen bond influences the mol­ecular conformation; the two benzene rings form a dihedral angle of 13.6 (3)°. In the crystal, inter­molecular N—H⋯O hydrogen bonds link the mol­ecules into chains along the *a* axis and weak inter­molecular C—H⋯O hydrogen bonds further link these chains into layers parallel to the *ac* plane.

## Related literature

For applications of hydrazone compounds, see: Hillmer *et al.* (2010 ▶); Raj *et al.* (2007 ▶). For the crystal structures of related hydrazone compounds, see: Vijayakumar *et al.* (2009 ▶); Liu & You (2010 ▶); Liu & Wang (2010 ▶). For related structures, see: Xu *et al.* (2009 ▶); Shafiq *et al.* (2009 ▶).
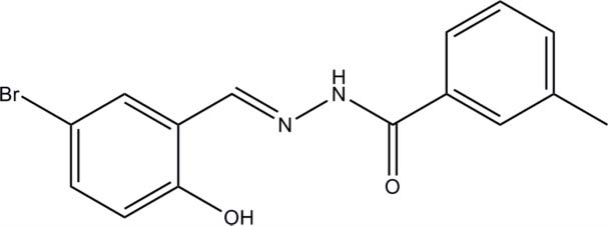


## Experimental

### 

#### Crystal data

C_15_H_13_BrN_2_O_2_
*M* *_r_* = 333.18
Monoclinic, 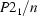

*a* = 7.138 (3) Å
*b* = 27.404 (10) Å
*c* = 7.859 (3) Å
β = 112.297 (5)°
*V* = 1422.4 (9) Å^3^
*Z* = 4
Mo *K*α radiation
μ = 2.89 mm^−1^
*T* = 298 K
0.13 × 0.10 × 0.10 mm

#### Data collection

Bruker SMART CCD area-detector diffractometer
Absorption correction: multi-scan (*SADABS*; Bruker, 2001 ▶) *T*_min_ = 0.705, *T*_max_ = 0.761
6807 measured reflections
3045 independent reflections
1489 reflections with *I* > 2σ(*I*)
*R*_int_ = 0.056

#### Refinement

*R*[*F* ^2^ > 2σ(*F* ^2^)] = 0.049
*wR*(*F* ^2^) = 0.106
*S* = 0.99
3045 reflections
185 parameters
1 restraint
H atoms treated by a mixture of independent and constrained refinement
Δρ_max_ = 0.32 e Å^−3^
Δρ_min_ = −0.36 e Å^−3^

### 

Data collection: *SMART* (Bruker, 2007 ▶); cell refinement: *SAINT* (Bruker, 2007 ▶); data reduction: *SAINT*; program(s) used to solve structure: *SHELXTL* (Sheldrick, 2008 ▶); program(s) used to refine structure: *SHELXTL*; molecular graphics: *SHELXTL*; software used to prepare material for publication: *SHELXTL*.

## Supplementary Material

Crystal structure: contains datablock(s) global, I. DOI: 10.1107/S1600536811025426/cv5119sup1.cif

Structure factors: contains datablock(s) I. DOI: 10.1107/S1600536811025426/cv5119Isup2.hkl

Supplementary material file. DOI: 10.1107/S1600536811025426/cv5119Isup3.cml

Additional supplementary materials:  crystallographic information; 3D view; checkCIF report

## Figures and Tables

**Table 1:** Hydrogen-bond geometry (Å, °)

*D*—H⋯*A*	*D*—H	H⋯*A*	*D*⋯*A*	*D*—H⋯*A*
N2—H2⋯O2^i^	0.90 (1)	2.02 (2)	2.890 (4)	163 (4)
O1—H1⋯N1	0.82	1.89	2.605 (4)	146
C14—H14⋯O1^ii^	0.93	2.44	3.226 (4)	143

Symmetry codes: (i) 
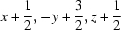
; (ii) 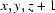.

**Fig. 1.**
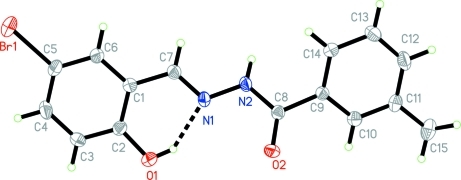
The molecular structure of (I). Displacement ellipsoids are drawn at the 30% probability level. Hydrogen atoms are shown as spheres of arbitrary radius and the intramolecular hydrogen bond is drawn as a dashed line.

**Fig. 2.**
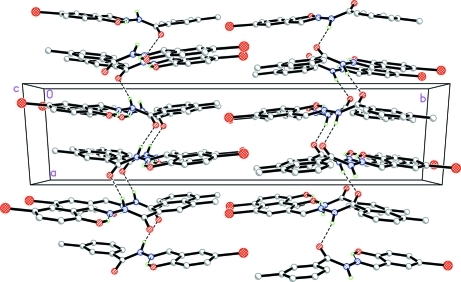
A portion of the crystal packing viewed along the c axis. N—H···O hydrogen bonds are shown as dashed lines. Hydrogen atoms not involved in N—H···O hydrogen bonding were omitted.

## supplementary crystallographic information

### Comment

Considerable attention has been focused on hydrazones and their medicinal applications (Hillmer *et al.*, 2010; Raj *et al.*, 2007). The crystal structures of such compounds are of particular interest (Vijayakumar *et al.*, 2009). As a continuation of our work with hydrazone compounds (Liu & You, 2010; Liu & Wang, 2010), we report herein the crystal structure of the title compound, (I).

In (I) (Fig. 1), two benzene rings are inclined with a dihedral angle of 13.6 (3) °. All the bond lengths are comparable to those observed in related structures (Xu *et al.*, 2009; Shafiq *et al.*, 2009), and those we reported previously (Liu & You, 2010; Liu & Wang, 2010).

In the crystal structure, molecules are linked through N–H···O hydrogen bonds (Table 1), to form one-dimensional chains running along the *a* axis (Fig. 2). Weak intermolecular C—H···O hydrogen bonds (Table 1)  link further these chains into layers parallel to *ac* plane.

### Experimental

The title compound was prepared by the condensation reaction of 5-bromosalicylaldehyde (0.05 mol, 10.0 g) and 3-methylbenzohydrazide (0.05 mol, 7.5 g) in anhydrous methanol (100 ml) at ambient temperature. Colourless block-shaped single crystals suitable for X-ray structural determination were obtained by slow evaporation of the solution for 5 d.

### Refinement

H2 was located from a difference Fourier map and refined isotropically, with the N–H distance restrained to 0.90 (1) Å. The remaining H atoms were positioned geometrically and constrained to ride on their parent atoms, with C–H distances of 0.93–0.96 Å, O–H distance of 0.82 Å, and with *U*_iso_(H) = 1.2*U*_eq_(C) and 1.5*U*_eq_(O1 and C15).

### Crystal data

**Table:**

C_15_H_13_BrN_2_O_2_	*F*(000) = 672
*M**_r_* = 333.18	*D*_x_ = 1.556 Mg m^−^^3^
Monoclinic, *P*2_1_/*n*	Mo *K*α radiation, λ = 0.71073 Å
Hall symbol: -P 2yn	Cell parameters from 983 reflections
*a* = 7.138 (3) Å	θ = 2.5–24.5°
*b* = 27.404 (10) Å	µ = 2.89 mm^−^^1^
*c* = 7.859 (3) Å	*T* = 298 K
β = 112.297 (5)°	Block, colourless
*V* = 1422.4 (9) Å^3^	0.13 × 0.10 × 0.10 mm
*Z* = 4	

### Data collection

**Table:**

Bruker SMART CCD area-detector diffractometer	3045 independent reflections
Radiation source: fine-focus sealed tube	1489 reflections with *I* > 2σ(*I*)
graphite	*R*_int_ = 0.056
ω scans	θ_max_ = 27.0°, θ_min_ = 2.9°
Absorption correction: multi-scan (*SADABS*; Bruker, 2001)	*h* = −9→8
*T*_min_ = 0.705, *T*_max_ = 0.761	*k* = −29→35
6807 measured reflections	*l* = −7→10

### Refinement

**Table:**

Refinement on *F*^2^	Primary atom site location: structure-invariant direct methods
Least-squares matrix: full	Secondary atom site location: difference Fourier map
*R*[*F*^2^ > 2σ(*F*^2^)] = 0.049	Hydrogen site location: inferred from neighbouring sites
*wR*(*F*^2^) = 0.106	H atoms treated by a mixture of independent and constrained refinement
*S* = 0.99	*w* = 1/[σ^2^(*F*_o_^2^) + (0.0322*P*)^2^ + 0.1905*P*] where *P* = (*F*_o_^2^ + 2*F*_c_^2^)/3
3045 reflections	(Δ/σ)_max_ < 0.001
185 parameters	Δρ_max_ = 0.32 e Å^−^^3^
1 restraint	Δρ_min_ = −0.36 e Å^−^^3^

### Special details

**Table:**

Geometry. All esds (except the esd in the dihedral angle between two l.s. planes) are estimated using the full covariance matrix. The cell esds are taken into account individually in the estimation of esds in distances, angles and torsion angles; correlations between esds in cell parameters are only used when they are defined by crystal symmetry. An approximate (isotropic) treatment of cell esds is used for estimating esds involving l.s. planes.
Refinement. Refinement of F^2^ against ALL reflections. The weighted R-factor wR and goodness of fit S are based on F^2^, conventional R-factors R are based on F, with F set to zero for negative F^2^. The threshold expression of F^2^ > 2sigma(F^2^) is used only for calculating R-factors(gt) etc. and is not relevant to the choice of reflections for refinement. R-factors based on F^2^ are statistically about twice as large as those based on F, and R- factors based on ALL data will be even larger.

### Fractional atomic coordinates and isotropic or equivalent isotropic displacement parameters (Å^2^)

**Table:**

	*x*	*y*	*z*	*U*_iso_*/*U*_eq_	
Br1	0.82155 (9)	1.017416 (16)	0.22011 (7)	0.0691 (2)	
N1	0.7539 (5)	0.78962 (11)	0.4049 (4)	0.0343 (8)	
N2	0.7822 (5)	0.76007 (11)	0.5550 (4)	0.0354 (8)	
O1	0.6966 (5)	0.80234 (9)	0.0601 (4)	0.0539 (9)	
H1	0.7095	0.7865	0.1525	0.081*	
O2	0.6037 (4)	0.69873 (9)	0.3697 (3)	0.0444 (8)	
C1	0.7856 (6)	0.86760 (13)	0.2857 (5)	0.0313 (9)	
C2	0.7363 (6)	0.85015 (13)	0.1057 (5)	0.0365 (10)	
C3	0.7251 (7)	0.88254 (15)	−0.0339 (5)	0.0474 (12)	
H3	0.6992	0.8709	−0.1518	0.057*	
C4	0.7520 (7)	0.93154 (15)	0.0010 (6)	0.0498 (12)	
H4	0.7406	0.9531	−0.0938	0.060*	
C5	0.7957 (6)	0.94894 (14)	0.1761 (6)	0.0430 (11)	
C6	0.8154 (6)	0.91721 (13)	0.3166 (5)	0.0386 (10)	
H6	0.8496	0.9293	0.4352	0.046*	
C7	0.8052 (6)	0.83446 (14)	0.4357 (5)	0.0358 (10)	
H7	0.8558	0.8460	0.5559	0.043*	
C8	0.7035 (6)	0.71426 (13)	0.5232 (5)	0.0327 (10)	
C9	0.7491 (6)	0.68420 (13)	0.6938 (5)	0.0320 (10)	
C10	0.7697 (6)	0.63410 (14)	0.6820 (5)	0.0367 (10)	
H10	0.7537	0.6202	0.5694	0.044*	
C11	0.8140 (6)	0.60430 (13)	0.8356 (6)	0.0386 (10)	
C12	0.8309 (6)	0.62636 (16)	1.0002 (6)	0.0514 (12)	
H12	0.8573	0.6069	1.1038	0.062*	
C13	0.8099 (7)	0.67597 (16)	1.0153 (6)	0.0498 (12)	
H13	0.8230	0.6897	1.1275	0.060*	
C14	0.7689 (6)	0.70533 (14)	0.8605 (5)	0.0400 (11)	
H14	0.7548	0.7389	0.8688	0.048*	
C15	0.8392 (8)	0.55033 (15)	0.8246 (7)	0.0644 (14)	
H15A	0.7782	0.5400	0.6987	0.097*	
H15B	0.7747	0.5340	0.8959	0.097*	
H15C	0.9808	0.5424	0.8722	0.097*	
H2	0.879 (5)	0.7674 (15)	0.664 (3)	0.080*	

### Atomic displacement parameters (Å^2^)

**Table:**

	*U*^11^	*U*^22^	*U*^33^	*U*^12^	*U*^13^	*U*^23^
Br1	0.0954 (5)	0.0325 (3)	0.0780 (4)	0.0030 (3)	0.0314 (3)	0.0053 (2)
N1	0.038 (2)	0.0305 (18)	0.034 (2)	0.0021 (15)	0.0127 (17)	0.0075 (14)
N2	0.037 (2)	0.0333 (19)	0.0296 (19)	−0.0013 (16)	0.0062 (17)	0.0078 (15)
O1	0.084 (2)	0.0348 (17)	0.0420 (17)	−0.0091 (16)	0.0230 (18)	−0.0069 (13)
O2	0.054 (2)	0.0402 (17)	0.0278 (16)	−0.0026 (13)	0.0030 (15)	−0.0019 (12)
C1	0.028 (3)	0.034 (2)	0.035 (2)	0.0047 (18)	0.0148 (19)	0.0009 (18)
C2	0.036 (3)	0.031 (2)	0.040 (3)	0.0017 (18)	0.012 (2)	−0.0017 (19)
C3	0.067 (4)	0.046 (3)	0.031 (2)	−0.002 (2)	0.020 (2)	−0.001 (2)
C4	0.060 (3)	0.046 (3)	0.045 (3)	0.005 (2)	0.021 (3)	0.016 (2)
C5	0.047 (3)	0.028 (2)	0.053 (3)	0.0012 (19)	0.017 (2)	0.0012 (19)
C6	0.040 (3)	0.034 (2)	0.036 (2)	0.0017 (19)	0.009 (2)	0.0013 (19)
C7	0.035 (3)	0.035 (2)	0.034 (2)	0.0006 (19)	0.010 (2)	0.0022 (18)
C8	0.032 (3)	0.031 (2)	0.034 (2)	0.0023 (18)	0.011 (2)	0.0027 (18)
C9	0.032 (3)	0.032 (2)	0.029 (2)	−0.0009 (17)	0.008 (2)	0.0021 (17)
C10	0.034 (3)	0.037 (2)	0.037 (2)	0.0035 (18)	0.012 (2)	0.0017 (18)
C11	0.031 (3)	0.034 (2)	0.052 (3)	0.0041 (19)	0.016 (2)	0.009 (2)
C12	0.050 (3)	0.056 (3)	0.041 (3)	−0.008 (2)	0.010 (2)	0.018 (2)
C13	0.059 (3)	0.057 (3)	0.033 (3)	−0.010 (2)	0.017 (2)	−0.001 (2)
C14	0.047 (3)	0.035 (2)	0.038 (2)	−0.005 (2)	0.016 (2)	−0.0043 (19)
C15	0.073 (4)	0.039 (3)	0.084 (4)	0.003 (2)	0.033 (3)	0.014 (2)

### Geometric parameters (Å, °)

**Table:**

Br1—C5	1.904 (4)	C6—H6	0.9300
N1—C7	1.279 (4)	C7—H7	0.9300
N1—N2	1.381 (4)	C8—C9	1.501 (5)
N2—C8	1.359 (4)	C9—C10	1.388 (5)
N2—H2	0.899 (10)	C9—C14	1.390 (5)
O1—C2	1.359 (4)	C10—C11	1.391 (5)
O1—H1	0.8200	C10—H10	0.9300
O2—C8	1.222 (4)	C11—C12	1.391 (5)
C1—C6	1.383 (5)	C11—C15	1.496 (5)
C1—C2	1.406 (5)	C12—C13	1.378 (6)
C1—C7	1.452 (5)	C12—H12	0.9300
C2—C3	1.390 (5)	C13—C14	1.394 (5)
C3—C4	1.369 (5)	C13—H13	0.9300
C3—H3	0.9300	C14—H14	0.9300
C4—C5	1.375 (5)	C15—H15A	0.9600
C4—H4	0.9300	C15—H15B	0.9600
C5—C6	1.371 (5)	C15—H15C	0.9600
			
C7—N1—N2	117.6 (3)	O2—C8—C9	122.6 (3)
C8—N2—N1	117.9 (3)	N2—C8—C9	114.1 (3)
C8—N2—H2	120 (3)	C10—C9—C14	120.0 (3)
N1—N2—H2	120 (3)	C10—C9—C8	118.4 (3)
C2—O1—H1	109.5	C14—C9—C8	121.6 (3)
C6—C1—C2	118.3 (3)	C9—C10—C11	121.2 (4)
C6—C1—C7	120.7 (3)	C9—C10—H10	119.4
C2—C1—C7	120.9 (3)	C11—C10—H10	119.4
O1—C2—C3	117.6 (4)	C10—C11—C12	117.6 (4)
O1—C2—C1	122.7 (3)	C10—C11—C15	121.5 (4)
C3—C2—C1	119.6 (3)	C12—C11—C15	120.9 (4)
C4—C3—C2	120.4 (4)	C13—C12—C11	122.4 (4)
C4—C3—H3	119.8	C13—C12—H12	118.8
C2—C3—H3	119.8	C11—C12—H12	118.8
C3—C4—C5	120.1 (4)	C12—C13—C14	119.2 (4)
C3—C4—H4	119.9	C12—C13—H13	120.4
C5—C4—H4	119.9	C14—C13—H13	120.4
C6—C5—C4	120.1 (4)	C9—C14—C13	119.7 (4)
C6—C5—Br1	120.4 (3)	C9—C14—H14	120.2
C4—C5—Br1	119.5 (3)	C13—C14—H14	120.2
C5—C6—C1	121.3 (4)	C11—C15—H15A	109.5
C5—C6—H6	119.3	C11—C15—H15B	109.5
C1—C6—H6	119.3	H15A—C15—H15B	109.5
N1—C7—C1	121.0 (3)	C11—C15—H15C	109.5
N1—C7—H7	119.5	H15A—C15—H15C	109.5
C1—C7—H7	119.5	H15B—C15—H15C	109.5
O2—C8—N2	123.3 (3)		

### Hydrogen-bond geometry (Å, °)

**Table:**

*D*—H···*A*	*D*—H	H···*A*	*D*···*A*	*D*—H···*A*
N2—H2···O2^i^	0.90 (1)	2.02 (2)	2.890 (4)	163 (4)
O1—H1···N1	0.82	1.89	2.605 (4)	146
C14—H14···O1^ii^	0.93	2.44	3.226 (4)	143

Symmetry codes: (i) *x*+1/2, −*y*+3/2, *z*+1/2; (ii) *x*, *y*, *z*+1.

## References

[bb1] Bruker (2001). *SADABS* Bruker AXS Inc., Madison, Wisconsin, USA.

[bb2] Bruker (2007). *SMART* and *SAINT* Bruker AXS Inc., Madison, Wisconsin, USA.

[bb3] Hillmer, A. S., Putcha, P., Levin, J., Hogen, T., Hyman, B. T., Kretzschmar, H., McLean, P. J. & Giese, A. (2010). *Biochem. Biophys. Res. Commun.* **391**, 461–466. 10.1016/j.bbrc.2009.11.080 PMC2812586 19914207

[bb4] Liu, S.-Y. & Wang, X. (2010). *Acta Cryst.* E**66**, o1775. 10.1107/S1600536810023937 PMC3006873 21587988

[bb5] Liu, S.-Y. & You, Z. (2010). *Acta Cryst.* E**66**, o1652. 10.1107/S1600536810022075 PMC3006755 21587880

[bb6] Raj, K. K. V., Narayana, B., Ashalatha, B. V., Kumari, N. S. & Sarojini, B. K. (2007). *Eur. J. Med. Chem.* **42**, 425–429. 10.1016/j.ejmech.2006.09.010 17074422

[bb7] Shafiq, Z., Yaqub, M., Tahir, M. N., Hussain, A. & Iqbal, M. S. (2009). *Acta Cryst.* E**65**, o2898. 10.1107/S1600536809044122 PMC2970999 21578480

[bb8] Sheldrick, G. M. (2008). *Acta Cryst.* A**64**, 112–122. 10.1107/S0108767307043930 18156677

[bb9] Vijayakumar, S., Adhikari, A., Kalluraya, B. & Chandrasekharan, K. (2009). *Opt. Mater.* **31**, 1564–1569.

[bb10] Xu, L., Huang, S.-S., Zhang, B.-J., Wang, S.-Y. & Zhang, H.-L. (2009). *Acta Cryst.* E**65**, o2412. 10.1107/S1600536809035764 PMC2970187 21577871

